# The GAs–RhMYB70 feedback loop fine-tunes cell expansion and petal size by modulating cellulose content in rose

**DOI:** 10.1093/hr/uhaf134

**Published:** 2025-05-21

**Authors:** Feifei Gong, Xiaoyu Wang, Qingcui Zhao, Dan Wang, Huijun Yan, Qigang Wang, Yiping Zhang, Yixin Zhang, Hongying Jian, Xianqin Qiu, Kaixue Tang, Hao Zhang, Weikun Jing

**Affiliations:** Beijing Key Laboratory of Development and Quality Control of Ornamental Crops, Department of Ornamental Horticulture, College of Horticulture, China Agricultural University, No. 2 Yuanmingyuan West Road, Haidian District, Beijing 100193, China; Flower Research Institute of Yunnan Academy of Agricultural Sciences, No. 2238 Beijing Road, Panlong District, Kunming, Yunnan 650205, China; Beijing Key Laboratory of Development and Quality Control of Ornamental Crops, Department of Ornamental Horticulture, College of Horticulture, China Agricultural University, No. 2 Yuanmingyuan West Road, Haidian District, Beijing 100193, China; Flower Research Institute of Yunnan Academy of Agricultural Sciences, No. 2238 Beijing Road, Panlong District, Kunming, Yunnan 650205, China; Flower Research Institute of Yunnan Academy of Agricultural Sciences, No. 2238 Beijing Road, Panlong District, Kunming, Yunnan 650205, China; Flower Research Institute of Yunnan Academy of Agricultural Sciences, No. 2238 Beijing Road, Panlong District, Kunming, Yunnan 650205, China; Flower Research Institute of Yunnan Academy of Agricultural Sciences, No. 2238 Beijing Road, Panlong District, Kunming, Yunnan 650205, China; Flower Research Institute of Yunnan Academy of Agricultural Sciences, No. 2238 Beijing Road, Panlong District, Kunming, Yunnan 650205, China; Flower Research Institute of Yunnan Academy of Agricultural Sciences, No. 2238 Beijing Road, Panlong District, Kunming, Yunnan 650205, China; Flower Research Institute of Yunnan Academy of Agricultural Sciences, No. 2238 Beijing Road, Panlong District, Kunming, Yunnan 650205, China; Flower Research Institute of Yunnan Academy of Agricultural Sciences, No. 2238 Beijing Road, Panlong District, Kunming, Yunnan 650205, China; Flower Research Institute of Yunnan Academy of Agricultural Sciences, No. 2238 Beijing Road, Panlong District, Kunming, Yunnan 650205, China; Flower Research Institute of Yunnan Academy of Agricultural Sciences, No. 2238 Beijing Road, Panlong District, Kunming, Yunnan 650205, China

## Abstract

Cell expansion in petals plays a crucial role in flower opening and final size in rose (*Rosa hybrida*), which largely determines its market value. While cell expansion is known to be closely associated with gibberellins (GAs), the underlying molecular mechanism remains elusive. Here, we measured the levels of GAs during flower opening and demonstrated that GA_3_ treatment significantly increases petal size. Moreover, we identified RhMYB70, an R2R3 MYB transcription factor, whose expression was inhibited by GA_3_ treatment. *RhMYB70* silencing resulted in larger petals and petal cell size than those of TRV control. Through transcriptome analysis and biochemical identification, RhMYB70 could directly bind to the promoter of the cellulose synthase gene *RhCESA8* and repress its transcription, thereby resulting in decreased cellulose content of petals and final size. In addition, we also identified the GA biosynthesis gene *RhGA3ox3* as an RhMYB70 target and demonstrated that RhMYB70 directly binds to and inhibits the promoter activity of *RhGA3ox3*, leading to decreased cellulose content of petals and petal size. Besides, knocking down *RhMYB70* expression not only resulted in increasing GA_1_ and GA_3_ levels in petals compared to TRV but also elevated cellulose content. Together, our findings reveal that the feedback regulation of GAs and RhMYB70 signaling fine-tunes cell expansion and petal size by modulating cellulose content of rose petals, providing genetic targets for improving rose flower quality.

## Introduction

Rose (*Rosa hybrida*) is one of the most valuable ornamental plants worldwide, widely used to produce cut flowers, as potted plants, and for landscaping and home gardens. The value of rose plants is highly susceptible to fluctuations in quality, with flower size being a key factor. Many studies have demonstrated that flower opening largely depends on the extent of petal expansion [1, 2]. Therefore, the mechanisms of flower opening, particularly petal expansion and its precise regulation, have remained a focal point for flower product quality control and an important topic for floral research.

Petal growth in rose is governed by the coordinated regulation of cell division and cell expansion. Cell expansion in plants involves cell wall degradation and resynthesis, changes in cell turgor, and cytoskeletal rearrangement [3]. Cellulose, a major component of the cell wall, forms the primary framework supporting the basic structure of the cell wall, providing plant cells with the ability to resist turgor pressure. Cellulose plays critical roles in cell division and differentiation, maintaining cell size and shape, and other aspects of plant development [4]. Deficiencies in the biosynthesis of cellulose or its components can lead to abnormal cell expansion [5]. Cellulose synthase (*CESA*) genes encode enzymes that are crucial for cellulose biosynthesis [4, 6, 7]. *AtCESA1*, *AtCESA,3*, and *AtCESA6* regulate cellulose biosynthesis in primary cell walls, whereas *AtCESA4*, *AtCESA7*, and *AtCESA8* are involved in secondary cell wall biosynthesis [8–10]. Silencing of *PhCESA3* in petunia (*Petunia hybrida*) leads to a reduction in cell size in the abaxial and adaxial epidermis of leaves and petals, accompanied by the expansion of internal cells [11]. Silencing of *RhCesA2* in rose petals also results in inhibited subepidermal cell expansion in petals [2].

GAs (gibberellins) are a phytohormone that promotes cell proliferation and expansion and regulates seed germination, leaf expansion, floral transition, stem and petiole elongation, and the development of flowers and fruits [12–16]. Among bioactive GAs, GA_1_, GA_3_, GA_4_, and GA_7_ play prominent roles. GA_3_ is a naturally occurring tetracyclic diterpene plant hormone and is the most widely used plant growth regulator, acting as a key regulator of various aspects of plant growth and development [13, 14]. The homeostasis of GAs in plants is regulated by multiple enzymes, with GA oxidases catalyzing both the biosynthetic and catabolic pathways. GA20ox and GA3ox are key rate-limiting enzymes in the GA biosynthetic pathway, while GA2ox plays a crucial role in the degradation of bioactive GAs. Together, these enzymes control the levels of GAs within the plant [17]. A deficiency of (or insensitivity to) GA results in dwarf phenotypes, while increased endogenous GA levels promote cell expansion and increase plant height [12, 18, 19]. When the extracellular GA concentration increases, active GA binds to GID1, and the receptor protein GID1 undergoes structural changes, allowing it to bind to DELLA protein and form GA/GID1/DELLA complex, promoting the degradation of DELLA protein by activated SCF, and ultimately releases the GA signal to exert physiological effects [15, 17]. In *R. hybrida*, silencing of the GA signaling gene *RhGAI1* enhances petal cell expansion, while silencing *RhNF-YC9* reduces GA content, leading to a decreased petal expansion rate [20, 21]. Overexpressing *GhWIP2* in *Gerbera hybrida* significantly reduces GA content, resulting in dwarf plants, smaller petals, and shorter sepals and petioles than in wild-type plants [22]. Despite these advances, however, the regulatory network underlying the role of GA in petal cell expansion remains unclear.

The MYB family of transcription factors constitutes one of the most extensive groups within the plant. The R2R3-MYB subfamily is the most prevalent. These proteins are characterized by two highly conserved MYB-DNA binding domains [23].

The R2R3-MYB family exhibits functional diversity and plays critical roles in plant growth and development, as well as regulating specific physiological processes. The R2R3-MYB transcription factor MYB56 influences seed size by regulating endosperm cell size in *Arabidopsis* [24]. Meanwhile, the MYB-like transcription factor DRMY1 regulates reproductive organ growth and size by modulating cell proliferation [25]. Overexpressing *PtMYB055* in transgenic poplar upregulates lignin biosynthesis genes, leading to an increase in secondary cell wall thickness [26]. The *Arabidopsis* MYB44, MYB70, MYB73, and MYB77, which share structural similarity and belong to the R2R3-MYB subfamily 22, have been extensively reported to be involved in the regulation of plant stress responses and phytohormone signaling pathways, including those of ABA, JA, and SA [19, 27]. In tomato, SlMYB70 has been shown to negatively regulate fruit ripening by directly repressing the transcription of ethylene biosynthesis genes [28]. MYB70 modulates root system development and seed germination in *Arabidopsis* by regulating the ABA and auxin signaling pathways, the balance of H_2_O_2_/O_2_, and suberization [29]. However, the role of MYB70 in the regulation of petal growth and development is still not fully understood.

In this study, we used data from a previous transcriptome analysis of rose petals treated with various phytohormones to determine that the R2R3-MYB subfamily gene *RhMYB70* is repressed by GA_3_ treatment. We elucidated the RhMYB70 vital roles in regulating cell expansion and petal size by affecting the GA levels and cellulose content of rose petals. Our findings reveal the role of the GAs–RhMYB70 feedback loop in the development of rose flowers, providing a robust theoretical foundation for precisely controlling rose petal size using plant growth regulators, with significant theoretical and practical implications.

## Results

### GA_3_ promotes cell expansion in rose petals

**Figure 1:**
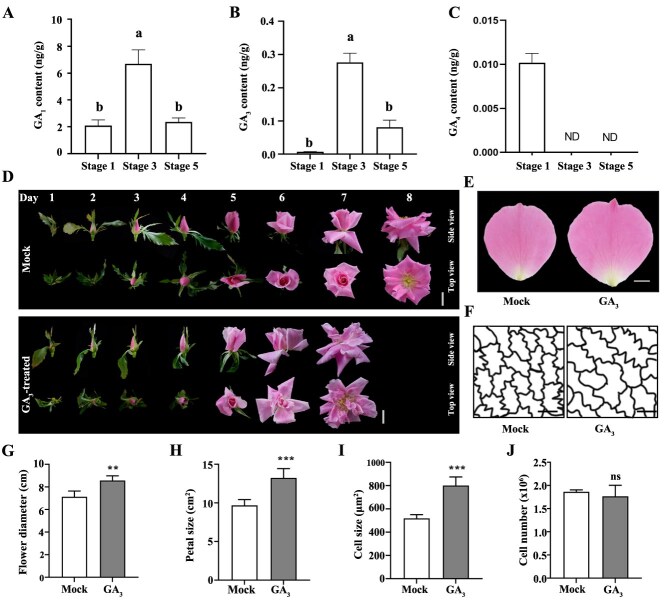
GA_3_ treatment increased petal size by promoting the cell expansion. C. Quantification of endogenous bioactive gibberellins (GAs) within petals at various developmental Stages. Different letters above the bars indicate significant differences according to Duncan’s multiple range test (*P* < 0.05), ND in C means not detected. D. Opening progression of rose flower treated with Mock and GA_3_. E. Phenotypes of the outermost layer petals in Mock and GA_3_-treated flower at Stage 5. F–J. Traces of abaxial subepidermis (AbsE) cells in the petal middle regions (F), flower diameters (G), petal size (H), average single AbsE cell areas (I), and cell number (J) in petals treated with Mock and GA_3_. Scale bars in D, E, and F represent 2 cm, 1 cm, and 20 μm, respectively. Data are presented as means ± SD (*n* = 3 in A–C; *n* = 5 in G, H, J; *n* = 10 in I). Statistical significance was determined using Student’s *t*-test, **P < 0.01 and ***P < 0.001. No significant difference is denoted by ns.

The size of petals are determined by the coordination between cell proliferation and cell expansion [1, 30]. The initiation of cell expansion in rose petals occurs at Stage 1, and Stage 1–3 is a period of rapid cell expansion, whereas Stage 3–5 primarily encompass significant alterations in petal angle [31]. As established in previous studies, GAs play a pivotal role in regulating petal cell expansion in rose (*R. hybrida*) [20, 26]. To characterize their dynamic involvement, we monitored the GA levels at different stages of flower opening, and found that GA_1_ and GA_3_ exhibited a sharp increase from Stage 1 to Stage 3, but significant decrease from Stage 3 to Stage 5 (Fig. 1A and B). However, the GA_4_ was not detected at Stage 3 and Stage 5. To further explore how GA regulates petal and flower size, we applied GA_3_ exogenously at Stage 1. GA_3_ significantly increased flower diameter (8.57 ± 0.41 cm for GA_3_ treatment vs 7.13 ± 0.51 cm for the Mock) and petal size (13.24 ± 1.20 cm^2^ for GA_3_ treatment and 9.67 ± 0.75 cm^2^ for Mock) (Fig. 1D, E, G, and  H). Measurements of abaxial epidermal cell size revealed that these cells were significantly larger under GA_3_ treatment (800.99 ± 73.57 μm^2^) than under Mock treatment (518.77 ± 31.73 μm^2^) (Fig. 1F and I). However, there was no significant difference in cell number at the fully open stage between Mock and GA_3_ treatments (Fig. 1J). These results indicate that GA might promote the cell expansion of petal cells.

### GA_3_ inhibits the transcription of *RhMYB70*

**Figure 2:**
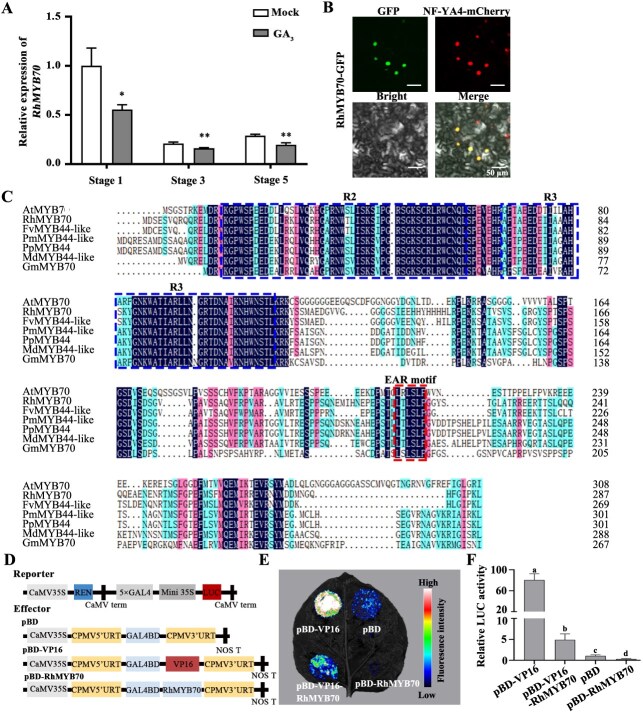
The characteristic analysis of RhMYB70. A. RT-qPCR analysis of *RhMYB70* transcript levels in petals treated with Mock and GA_3_ at Stage 1, Stage 3, Stage 5. *RhUBI2* was used as an internal control. B. Multiple sequence alignment of amino acid sequence of RhMYB70 protein with other six species MYB proteins. C. Subcellular localization of RhMYB70 in *N. benthamiana* leaves by using NF-YA4-mCherry as the nuclear marker. Scale bars represent 50 μm. D–F. Transcriptional repressor activity of RhMYB70. Schematic representation of the reporter and effector constructs was shown in D; live imaging of *N. benthamiana* leaves of RhMYB70 transcriptional activity in E; quantitative analysis of RhMYB70 transcriptional repressor activity was in F. Data are shown as means ± SD (*n* = 3 in A and F). Different lowercase letters (F) indicate significant differences according to one-way ANOVA with Duncan’s multiple comparisons test (*P* < 0.05). Asterisks (in A) indicate statistically significant differences (**P < 0.05; **P < 0.01) by two-sided Student’s *t*-test.

Based on previously reported transcriptome sequencing data [32], we determined that the expression of RchiOBHmChr6g0255341 was significantly repressed by GA_3_ (Supplementary Fig. S1). Reverse transcription-quantitative polymerase chain reaction (RT-qPCR) further confirmed that RchiOBHmChr6g0255341 expression was inhibited by GA_3_ and its expression levels decreased from Stages 1 to 3 (Fig. 2A). Phylogenetic examination coupled with conserved domain alignment indicated that the protein produced by the RchiOBHmChr6g0255341 gene is a member of the R2R3-MYB family subgroup 22, which is involved in response to both biotic and abiotic stresses [33], and is closely related to *Arabidopsis* MYB70 (AT2G23290) (Supplementary Fig. S2 and Fig. 2B). We thus named this protein RhMYB70. The fusion vector of RhMYB70 protein emits fluorescence exclusively in the nucleus, indicating that it functions within this cellular compartment (Fig. 2B). A transcriptional activity assay revealed that RhMYB70 is a transcriptional repressor (Fig. 2D–F).

### RhMYB70 directly represses *RhCESA8* expression and reduced cellulose content of rose petals

**Figure 3:**
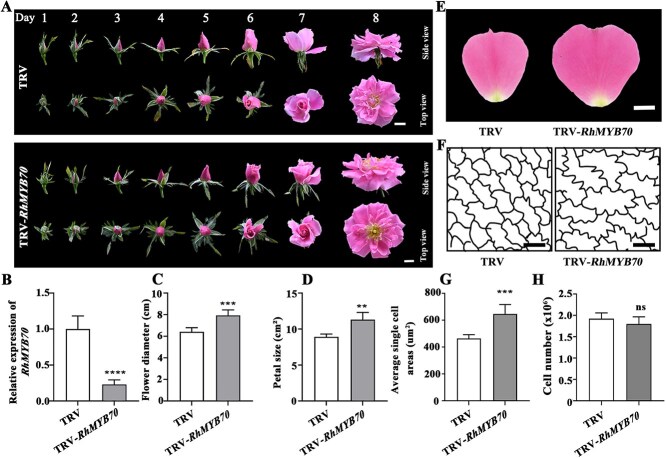
*RhMYB70* regulates cell expansion and petal size by modulating cellulose content of rose petals. A. Flower phenotypes of TRV control and TRV-*RhMYB70* plants. B. RT-qPCR showing the *RhMYB70* expression in TRV control and *RhMYB70*-silenced plants. C–H. Flower diameter (C), petal size data (D) and photos (E), traces of AbsE cell outlines in the middle regions of petals (F), average single AbsE cell areas (G), and cell number (H) of petals in TRV and TRV-*RhMYB70* flower at Stage 5. Scale bars represent 1 cm in A & E, 20 μm in F. Mean values ± SD are shown (*n* = 5 in B, C, and D; *n* = 10 in G; and *n* = 5 in H). Asterisks, significant differences by using two-sided Student’s *t*-test (**P < 0.01; ***P < 0.001; ****P < 0.0001; ns, no significant difference).

To elucidate the function of RhMYB70 in the expansion of petal cells, we silenced *RhMYB70* using virus-induced gene silencing (VIGS). Notably, *RhMYB70* silencing led to a larger flower diameter (7.93 ± 0.38 cm) and petal size (11.30 ± 0.38 cm^2^) than those in the TRV control (flower diameter, 6.41 ± 0.50 cm; petal size, 8.92 ± 1.01 cm^2^) (Fig. 3A–E). The cell size in petals from the *RhMYB70*-silenced lines (646.91 ± 69.55 μm^2^) was larger than that in petals from the TRV lines (464.66 ± 27.92 μm^2^) (Fig. 3F–G). These results suggested that RhMYB70 might regulate cell expansion and petal size in a GA-dependent manner.

**Figure 4:**
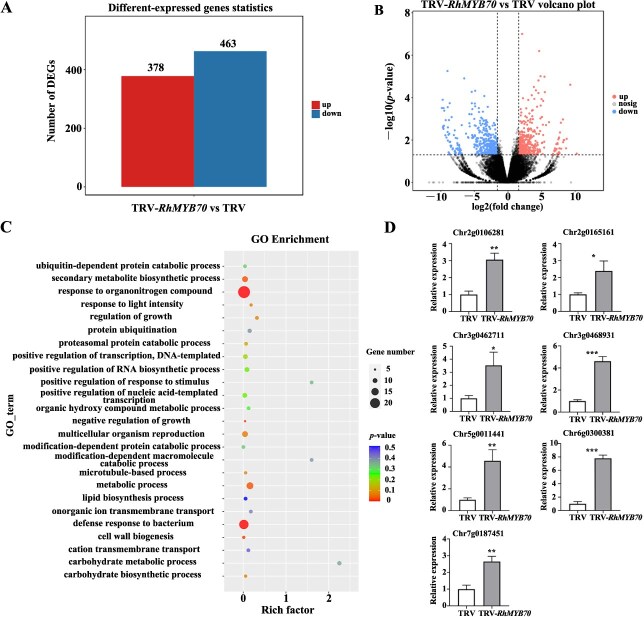
RhMYB70 influences expression of genes related to cell wall biogenesis in rose petals. A. The number of upregulated genes and downregulated genes. B. Scatterplots of gene expression in *RhMYB70*-silenced petals relative to the TRV control. Blue and red dots represent downregulated and upregulated genes, respectively. C. Significantly enriched GO enrichment analysis between TRV-*RhMYB70* and TRV control. D. qRT-PCR analysis of DEGs involved in cell wall biogenesis terms in TRV control and TRV-*RhMYB70* flower at Stage 5. Asterisks, significant differences by using two-sided Student’s *t*-test (**P < 0.01; ***P < 0.001; ****P < 0.0001; ns, no significant difference).

To better investigate the possible regulatory network of petal growth involving RhMYB70, we performed RNA-sequencing analysis (RNA-seq) of TRV and TRV-*RhMYB70*. We identified 841 differentially expressed genes (DEGs), comprising 378 upregulated genes and 463 downregulated genes (Fig. 4A). Several DEGs were enriched in the Gene Ontology (GO) terms ‘cell wall biogenesis’, ‘secondary metabolite biosynthetic process’, ‘metabolic process’, and ‘negative regulation of growth’ (Fig. 4C, Supplementary Table S1). Cell wall biogenesis is closely related to cell expansion [2, 20, 21, 31]. Indeed, we identified seven DEGs (RchiOBHmChr7g0187451, RchiOBHmChr3g0462711, RchiOBHmChr2g0106281, RchiOBHmChr6g0300381, RchiOBHmChr3g0468931, RchiOBHmChr5g0011441, and RchiOBHmChr2g0165161) enriched in this term among the DEGs (Supplementary Table S1). We measured the expression of the cell wall biogenesis genes in TRV and TRV-*RhMYB70* petals by RT-qPCR. Seven genes were significantly upregulated in TRV-*RhMYB70* petals compared to the TRV (Fig. 4D).

**Figure 5:**
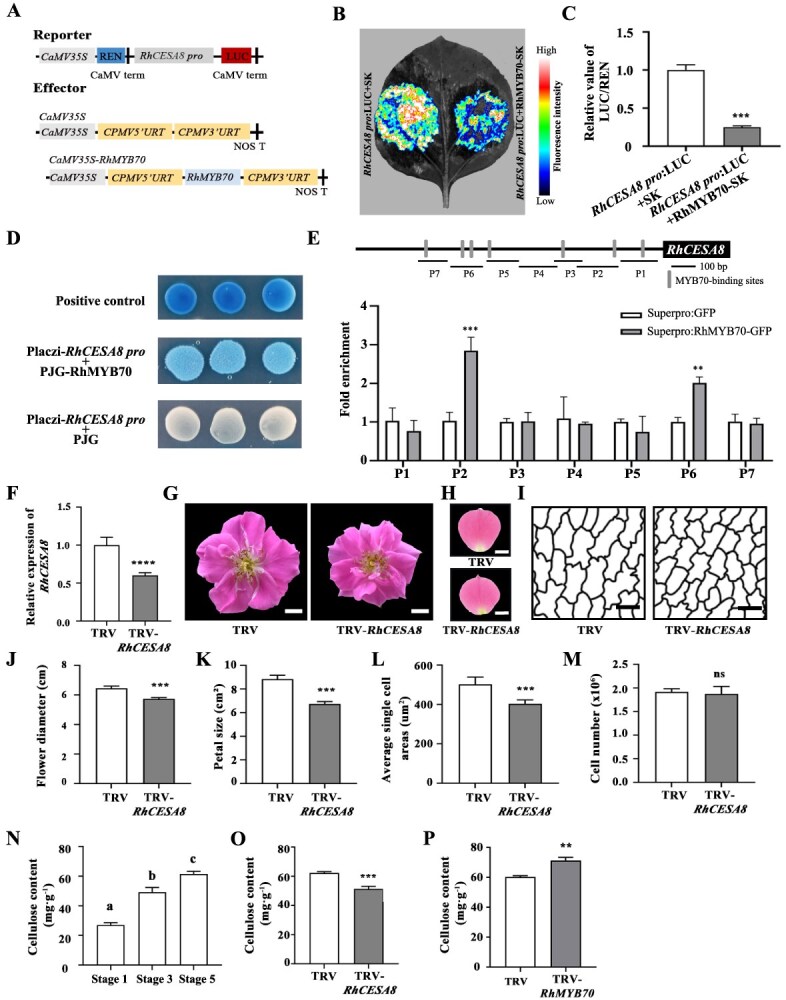
RhMYB70 directly binds to the *RhCESA8* promoter and represses its transcriptional activity. A–C. Effect of RhMYB70 on *RhCESA8* promoter. Effector and reporter constructs (A); imaging of luciferase activity in *N. benthamiana* leaves (B); quantitative analysis of LUC/REN of RhMYB70 on *RhCESA8* promoter (C). D. Y1H assay showing the binding of RhMYB70 to the *RhCESA8* promoter. E. ChIP-qPCR analysis of RhMYB70 binding to P2 and p6 fragments of the *RhCESA8* promoter. F. RT-qPCR showing the expression of *RhCESA8* in TRV control and *RhCESA8*-silenced plants. G. The flowers in TRV and TRV-*RhCESA8* plants at the fully opened Stage 5. Scale bars, 1 cm. H. The petals in TRV and TRV-*RhCESA8* plants at the fully opened Stage 5. Scale bars, 1 cm. I. Traces of AbsE cell outlines of TRV control and *RhCESA8*-silenced plants in the middle regions of petals at Stage 5. Scale bars, 20 μm. J & K. Flower diameter (J) and petal size (K) of TRV control and *RhCESA8*-silenced plants at Stage 5. L & M. Average single AbsE cell areas (L) and cell number (M) of TRV control and *RhCESA8*-silenced petals at Stage 5. N. The cellulose content at Stage 1, Stage 3, and Stage 5.0. The cellulose content of TRV control and Rh*MYB70*-silenced plants at Stage 5. P. The cellulose content of TRV control and *RhCESA8*-silenced plants at Stage 5. Mean values ± SD are shown (*n* = 5 in C, E, F, J, K, M, N, O, and P; *n* = 3 in F; *n* = 10 in L;) Asterisks represent statistically significant differences by using two-sided Student’s *t*-test (**P < 0.01; ***P < 0.001; ****P < 0.0001).

To investigate whether RhMYB70 directly binds to these gene promoters to regulate their expression, we conducted several experiments. Moreover, yeast one-hybrid (Y1H) assays further indicated that RhMYB70 binds to the promoter of *RchiOBHmChr5g0011441* but not to those of the other genes examined (Fig. 5D, Supplementary Fig. S4). We analyzed the transcriptome data of roses treated with GA_3_ and determined that GA_3_ treatment significantly increases *RchiOBHmChr5g0011441* transcript levels (Supplementary Fig. S3A). Phylogenetic analysis and conserved domain alignment showed that RchiOBHmChr5g0011441 encodes a cellulose synthase, which is closely related to *Arabidopsis* CESA8 (AT4G18780). We therefore named this protein RhCESA8 (Supplementary Fig. S3B). Dual-luciferase assays and chromatin immunoprecipitation qPCR (ChIP-qPCR) demonstrated that RhMYB70 binds to the P2 and P6 regions of the *RhCESA8* promoter and represses its transcriptional activity (Fig. 5A, B, C, E).

Furthermore, silencing of *RhCESA8* resulted in smaller flower diameter (5.74 ± 0.09 cm), petal size (6.74 ± 0.17 cm^2^), and cell size (403.66 ± 18.41 μm^2^) than those of TRV control (flower diameter, 6.47 ± 0.15 cm; petal size, 8.84 ± 0.75 cm^2^; cell size, 502.09 ± 35.29 μm^2^) (Fig. 5F–M). As RhCESA8 role as a key enzyme for cellulose biosynthesis, we first detected the dynamic changes of cellulose and found the accumulation levels of cellulose gradually increased during flower opening, implying that it is closely related to cell expansion (Fig. 5N). We further revealed that the cellulose content in *RhCESA8*-silenced petals was significantly less than that of TRV (Fig. 5O). Besides, the cellulose content in *RhMYB70*-silenced petals increased significantly compared to the TRV, with a 20.30% rise (Fig. 5P). Taken together, these results demonstrated that GAs regulated cell expansion and petal size by repressing *RhMYB70* expression, thereby activating *RhCESA8* expression and raising the cellulose content of rose petals.

### RhMYB70 also represses *RhGA3ox3* expression and regulates GA biosynthesis in rose petals

**Figure 6:**
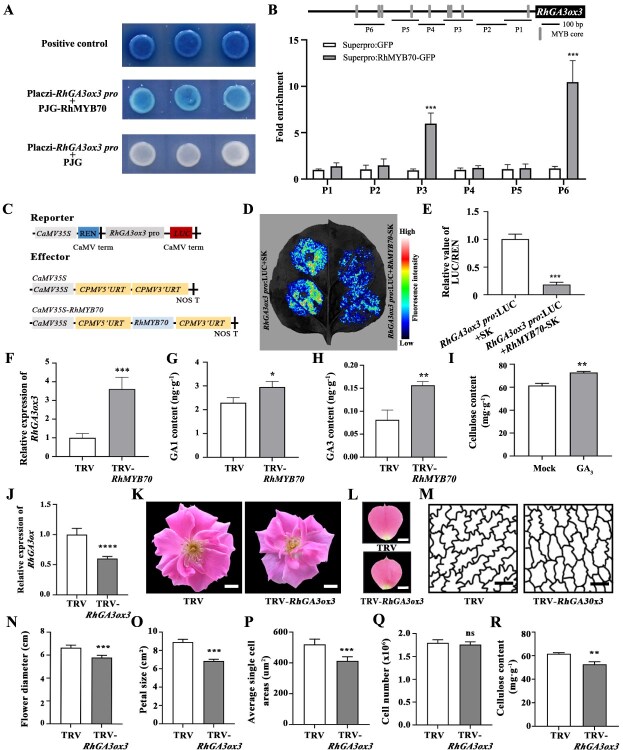
RhMYB70 represses *RhGA3ox3* expression by directly binding to its promoter. A. Y1H analysis indicated that RhMYB70 directly binds to the *RhGA3ox3* promoter. B. ChIP-qPCR analysis of RhMYB70 binding to P2 and p6 fragments of the *RhGA3ox3* promoter. C–E. Effect of RhMYB70 on *RhGA3ox3* promoter. Effector and reporter constructs (C); imaging of luciferase activity in *N. benthamiana* leaves (D); quantitative analysis of LUC/REN of RhMYB70 on *RhGA3ox3* promoter (E). F. RT-qPCR analysis of *RhGA3ox3* expression in TRV control and *RhMYB70*-silenced flower petals. G and H. GA_1_ and GA_3_ contents of TRV and TRV-*RhMYB70* petals. I. The cellulose content in Mock and GA_3_-treated petals at Stage 5. J. RT-qPCR showing the expression of *RhGA3ox3* in TRV control and *RhGA3ox3*-silenced plants. K. The flowers in TRV and TRV-*RhGA3ox3* plants at the fully opened Stage 5. Scale bars, 1 cm. L. The petals in TRV and TRV-*RhGA3ox3* plants at the fully opened Stage 5. Scale bars, 1 cm. M. Traces of AbsE cells outlines of TRV control and *RhGA3ox3*-silenced plants in the middle regions of petals at Stage 5. Scale bars, 20 μm. N and O. Flower diameter (N) and petal size (O) of TRV control and *RhGA3ox3*-silenced plants at Stage 5. P and Q. Average single AbsE cell areas (Q) and cell number (R) of TRV control and *RhGA3ox3*-silenced petals at Stage 5. R. The cellulose content of TRV control and *RhGA3ox3*-silenced plants at Stage 5. Data are shown as means ± SD (*n* = 3 in B, G–J, N, Q, and R; *n* = 5 in E and F; *n* = 10 in P). Asterisks represent statistically significant differences by using two-sided Student’s *t*-test (*P < 0.05; **P < 0.01; ***P < 0.001; ****P < 0.0001).

Notably, function annotation analysis of the DEGs identified *RchiOBHmChr1g0355231*, which is involved in GA biosynthetic and catabolic processes (Supplementary Table S2, Supplementary Fig. S5); this gene is annotated as *RhGA3ox3*. To examine whether RhMYB70 directly regulates *RhGA3ox3* expression, we conducted a Y1H assay and determined that RhMYB70 could bind to the *RhGA3ox3* promoter (Fig. 6A). ChIP-qPCR and dual-luciferase reporter assays suggested that RhMYB70 binds to P3 and P6 regions of the *RhGA3ox3* promoter and inhibits its transcriptional activity (Fig. 6B–E). Moreover, silencing of *RhMYB70* significantly increased *RhGA3ox3* transcript levels, as revealed by RT-qPCR (Fig. 6F). As *RhGA3ox3* is an important GA biosynthesis gene, we also examined the GA levels in TRV and TRV-*RhMYB70* petals. GA_1_ and GA_3_ levels were higher in *RhMYB70*-silenced petals than in TRV controls (Fig. 6G and H). We further discovered that the cellulose content in GA_3_-treated rose petals was higher than that in the control group (Fig. 6I).

**Figure 7:**
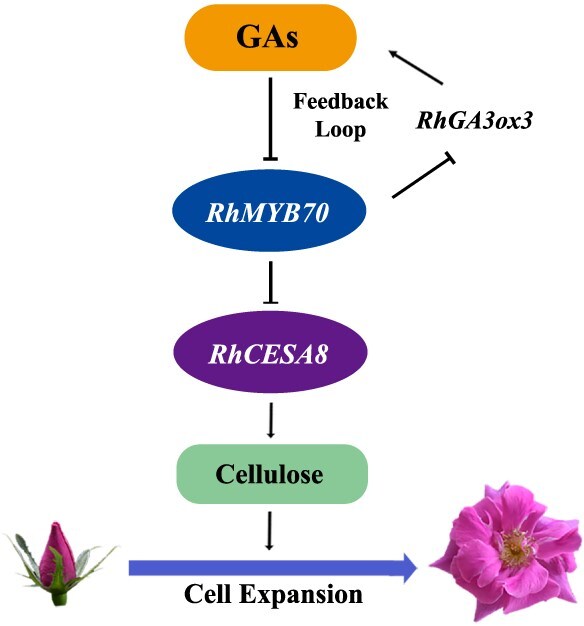
The assumed model of feedback loop of GAs and RhMYB70 in rose petals. We hypothesize that during the early flower opening process (Stage 1–3), higher levels of GAs significantly inhibit RhMYB70 expression, which acts as a negative regulator of cell expansion and petal size. Decreasing *RhMYB70* abundance enhances *RhCESA8* expression, thereby increasing the cellulose content of petals and promoting cell expansion. In addition, slightly elevated *RhMYB70* abundance at Stage 5 represses the transcriptional level of *RhGA3ox3*, thus maintaining relatively lower GA content at later opening stages. The feedback loop between GAs and RhMYB70 fine-tunes both *RhCESA8* expression and the cellulose content of petals, ultimately determining the sizes of petals and flowers in rose plants.

Furthermore, silencing of *RhGA3ox3* also resulted in smaller flower diameter (5.80 ± 0.18 cm), petal size (6.85 ± 0.16 cm^2^), and cell size (412.19 ± 26.91 μm^2^) than those of the TRV control (flower diameter, 6.65 ± 0.23 cm; petal size, 8.92 ± 0.27 cm^2^; cell size, 520.06 ± 32.92 μm^2^) (Fig. 6R). Based on these findings, the feedback loop of GA and RhMYB70 signaling fine-tunes *RhCESA8* expression and cellulose content, ultimately determining petal and flower size in rose (Fig. 7). Our findings provide important insights into the GA-mediated network regulating cell expansion and organ size in rose.

## Discussion

GAs are crucial in various aspects of plant growth and development. They significantly influence processes such as seed germination, the formation of root structures, elongation of stems, development of flower organs, and the expansion of fruit [16, 34–36]. Many studies have revealed the critical roles of GA in regulating petal cell expansion in rose. Silencing of the DELLA gene *RhGAI1* promotes petal cell expansion: RhGAI1 interacts directly with the promoter region of the cellulose synthase gene *RhCESA2*, thereby repressing its transcription and consequently limiting petal cell enlargement [20]. The transcription factor gene *RhNF-YC9* is repressed by ethylene, and silencing of *RhNF-YC9* reduces the expression of the GA biosynthesis gene *RhGA20ox* and increases the expression of the GA inactivation gene *RhGA2ox*, leading to reduced active GA levels and ultimately slowing cell expansion and petal growth rates [21]. In this study, exogenous application of GA_3_ treatment during flower Stage 1 can induce petal cell enlargement, resulting in an increase in the final petal area, and the obtained data are consistent with previous studies. Previous studies have demonstrated that GA promotes petal cell expansion and flower opening in roses by regulating the expression of genes related to cell turgor modulation (like PIP1;1/2;1), cell wall loosening (CesA2/3, EXPA1/8, XTH2/6/30), and cytoskeleton remodeling (TOR3/4) [20, 21]. However, our study specifically revealed that GA_3_ accelerates rose flower opening through the MYB-CESA module. We proposed that the role of GAs on flower opening may be more vigorous than the impact of *RhMYB70* silencing alone, resulting in flowers treated with GA_3_ opening 1 day earlier than those treated with TRV or the Mock.

The R2R3-MYB class of transcription factors constitutes a vast and ubiquitous family of regulatory proteins in the plant kingdom [23]. These factors are crucial in a spectrum of physiological activities, such as secondary metabolism, phytohormone signaling, and responses to environmental stress [37, 38]. RhMYB108 orchestrates signals from ethylene and jasmonic acid during petal aging [39], whereas RhMYB1 is engaged in the biosynthesis of fragrance and pigments in *R. hybrida* [40]. RhMYB123 and RhMYB17 have been implicated in cold-mediated floral organ development [41, 42] and RhMYB73 regulates cytokinin levels, influencing the transition from cell division to cell expansion, thereby affecting the shift from cell proliferation to expansion [30]. Plant R2R3-MYB transcription factors are known to bind to specific *cis*-regulatory elements, which are the MYB core sequence ‘YNGTTR’ and the AC element ‘ACCWAMY’ [43, 44]. Based on this, we predicted the binding elements in the promoters of *RhCESA8* and *RhGA3ox3*. Through ChIP-qPCR validation, we confirmed that the RhMYB70 protein can directly bind to the promoters of *RhCESA8* and *RhGA3ox3*. This finding is consistent with the reported MYB70-binding sites in the promoter regions of downstream genes *GH3.3* and *PER57* in *Arabidopsis* [29]. To date, research on the MYB70 gene, a member of the R2R3-MYB subfamily 22, remains limited. In the present study, we uncover a novel feedback regulatory module involving GAs and RhMYB70 signaling that fine-tunes cell expansion and petal size in rose. Our findings demonstrate that *RhMYB70* expression is inhibited by GA₃. Silencing of *RhMYB70* results in increased GA levels in petals, enhanced expansion of abaxial epidermal cells, and larger petal size. These results suggest that RhMYB70 may act as a key regulator within the GA signaling pathway that modulates petal expansion.

Cell division and expansion during plant growth are closely associated with the biosynthesis of cell wall components. This enlargement is facilitated by the cell wall’s loosening, which allows for a swift increase in size due to turgor pressure, a mechanism that is contingent upon the relaxation of the cell wall, alterations in turgor pressure, and the restructuring of the cytoskeleton [3]. A number of R2R3-MYB transcription factors have been identified to modulate the formation of the secondary cell wall, either directly or indirectly, in *Arabidopsis* [23, 45]. Kim *et al*. have shown that MYB46 has a direct influence on the expression of cellulose synthase genes *CESA4*, *CESA7*, and *CESA8*, which results in the abnormal accumulation of secondary cell walls [46, 47]. NST1 and SND1 are known to enhance the activity of cellulose synthase genes *IRX3/CESA7* and *CESA8* [48, 49]. Furthermore, the overexpression of SND1-related genes *PtrWND2B* and *PtrWND6B* in poplar (*Populus trichocarpa*) triggers the expression of CESA4, CESA7, and CESA8, leading to the abnormal accumulation of secondary cell walls [50]. In cotton (*Gossypium hirsutum*), GhCESA8 has been recognized as a pivotal element in the biosynthesis of cellulose within secondary cell walls, potentially impacting the cellulose content and the strength of the fibers [51]. Similarly, in our study, it was clearly demonstrated that the silencing of *RhCESA8* significantly reduced the cellulose content in petals, leading to petals become smaller.

Gibberellins modulate petal size by regulating the expansion of petal cells. Cell expansion is a dynamic process that necessitates continuous remodeling of the cell wall to accommodate the increasing cell volume [52]. Cellulose, a major component of the cell wall, provides structural support and stability during cell expansion and collaborates with other cell wall components to maintain the dynamic equilibrium of the cell wall [52]. In *Arabidopsis*, GA-related mutants exhibit significant alterations in the expression of CESA genes and in cellulose synthesis [53]. The CESA genes from *Arabidopsis* are upregulated in response to GA induction, and their promoter regions contain at least one GAMYB element [54]. The GAMYB transcription factors, which belong to a distinct subclass of the MYB superfamily, are widely present in plant species and many members of this subclass play regulatory roles in cell wall biosynthesis [55–57]. In this study, we identified a GAMYB transcription factor, RhMYB70. Our results show that both GA_3_ treatment and the suppression of RhMYB70 lead to a marked increase in the transcriptional level of the cellulose synthase gene *RhCESA8*, thereby elevating cellulose content. Biochemical assays further confirmed that RhMYB70 directly interacts with the promoter region of *RhCESA8* to regulate its expression. Additionally, in rose petals with silenced *RhGA3ox3*, cellulose content is significantly reduced, resulting in smaller petals. While our study has shown that RhMYB70 directly influences the expression of a gene involved in GA biosynthesis (*RhGA3ox3*) and a gene related to cell wall restructuring (*RhCESA8*), thereby affecting GA concentrations and cell wall composition. However, further investigation is needed to understand the precise mechanisms by which GAs and RhMYB70 coordinate the cell wall’s loosening and the expansion of petals in rose plants.

## Materials and methods

### Plant materials and growth conditions

The acquisition and culture of sterile seedlings of *R. hybrida* ‘Samantha’ were described previously [30]. Vigorous shoots of rose seedlings with bud points were used as explants and subjected to disinfection treatment. Stems containing a single node were cultured on propagation medium containing 4.4 g/l MS salts supplemented with 30 g/l sucrose, 1.0 mg/l 6-benzylaminopurine (6-BA), 0.05 mg/l naphthaleneacetic acid (NAA), 3.0 mg/l GA_3_, pH 5.9, and 6 g/l agar. After 30 days, the plantlets were transferred to rooting medium containing the same concentration of MS salts, 30 g/l sucrose, 0.1 mg/l NAA, pH 5.9, and 7 g/l agar. After 30 more days of growth, the rooted plantlets were transplanted into 9-cm-diameter pots filled with a 1:1 (v/v) mixture of peat soil and vermiculite.

Plants were cultivated in a greenhouse with stringent environmental parameters: a 16-h light/8-h dark cycle, temperatures maintained at 22 ± 1°C, and a humidity level of 50%. The lighting was supplied by 16 W fluorescent lamps, providing an intensity of 100 μmol m^−2^ s^−1^. *Nicotiana benthamiana* was grown under identical environmental conditions as the rose seedlings.

### Exogenous GA_3_ treatment

GA_3_ treatment was performed as described previously [30]. Briefly, the rose flower buds at Stage 1 (unopened bud with visible petals) were treated with a 100 μM GA_3_ solution, applied every 48 h, continuing until the flowers reached full blooming; for comparison, control blossoms used an equivalent volume of water solution.

### Quantification of endogenous GA levels

A 50-mg petal sample was extracted with a MeOH/H_2_O/formic acid mixture (15:4:1 v/v/v), vortexed, and centrifuged. The supernatant was evaporated, and the residue was treated with formic acid, water, and ethyl acetate, then reextracted with ethyl acetate. The combined fractions were evaporated, reconstituted in ACN, and treated with triethylamine and 3-bromopropyltrimmethylammonium bromide. After incubation and evaporation, the sample was redissolved in ACN/H_2_O, filtered, and analyzed by LC–MS/MS (Liquid Chromatography Tandem Mass Spectrometry ) using the AB Sciex QTRAP® 6500+ platform.

### Microscopic observation and cell counting

When a flower reached Stage 5, the flower’s petals become fully expanded, facilitating the measurement of the flower diameter with a digital caliper. Petal images were captured digitally using a flatbed scanner to measure total petal area with ImageJ software (National Institutes of Health, USA). The outer petals were meticulously excised and preserved in formaldehyde-acetic acid (FAA) solution. The fixed petals were decolorized using a series of ethanol treatments.

For microscopic analysis, the adaxial epidermal cells were observed under an optical microscope. Specific cells were chosen from the central region of the petal for detailed examination and photography. Statistics on the number and size of petal cells were executed in accordance with the procedure detailed by Jing, Jin & Gong *et al* [30, 58–60].

### Virus-induced gene silencing

The VIGS technique was executed following the previously outlined protocol [61]*.* Gene-specific fragments for *RhMYB70* (412 bp), *RhCESA8* (355 bp), and *RhGA3ox3* (252 bp) were amplified and inserted into the pTRV2 vector to obtain pTRV2::*RhMYB70*， pTRV2::*RhCESA8*, pTRV2::*RhGA3ox3*. pTRV1, pTRV2, pTRV2::*RhMYB70,* pTRV2::*RhCESA8*, and pTRV2::*RhGA3ox3* were introduced into the *Agrobacterium tumefaciens* GV3101 strain. These were then cultured in an LB medium supplemented with kanamycin and rifampicin. The *Agrobacterium* cells, after being cultured, underwent centrifugation and were subsequently resuspended in a buffer solution. The OD_600_ of the resuspended bacterial solution was adjusted to 1.0, and the resuspended pTRV2, pTRV2-*RhMYB70,* pTRV2-*RhCESA8*, and pTRV2-*RhGA3ox3* bacterial solutions were mixed with pTRV1 bacterial solution at equal volumes. The mixed bacterial solution was incubated in a dark environment for 3–5 h.

*In vitro-*grown rose seedlings showing comparable growth were submerged in the *Agrobacterium* suspension and subjected to vacuum infiltration. Following infiltration, the seedlings were incubated in a dark environment at a low temperature of 8°C for 3 days. Post-incubation, seedlings were placed in pots with a 1:1 peat-vermiculite mix and grown in the cultivation room. The developmental progression of the rose flower was meticulously recorded with photographs at the stages of flowering. The primers employed in the VIGS assay are detailed in Supplementary Table S3.

### Subcellular localization

The *RhMYB70* coding sequence was fused with GFP (Green Fluorescent Protein ) in the *pSuper::RhMYB70* vector, with NF-YA4-mCherry as a nuclear marker [62]. Constructs were introduced into *A. tumefaciens* GV3101, mixed, and co-infiltrated into *N. benthamiana* leaves. After 3 days, leaves were analyzed with an Olympus FV3000 confocal microscope, using 488 nm for GFP and 561 nm for mCherry excitation.

### RNA-Seq

High-quality RNA was extracted from rose petals, both from pTRV2- and *RhMYB70*-silenced samples, utilizing the hot borate method to ensure suitability for sequencing [1]. For each sample, cDNA libraries were synthesized from 1 μg of total RNA. These libraries were prepared in accordance with established protocols and sequenced using an Illumina sequencing platform. The bioinformatics analyses encompassed sequencing, data processing, assembly, and annotation. The RNA-seq reads were aligned to the reference genome of *Rosa chinensis* ‘Old Blush’ (GenBank ID 8255808) and the data was archived in the NCBI BioProject database with the accession number PRJNA1162946.

Differential gene expression was analyzed with log_2_(fc) ≥ 1.5 or ≤ −1.5 and *P*-value ≤ 0.05. Differentially expressed genes underwent GO and KEGG (Kyoto Encyclopedia of Genes and Genomes) analyses. Each sample had three biological replicates.

### RNA extraction and RT-qPCR

The process of isolating total RNA from rose petals was accomplished through the application of the hot borate extraction method [1]. Following this, the synthesis of cDNA was initiated using 1 μg of the extracted RNA, strictly following the protocol outlined in the HiScript III All-in-one RT SuperMix kit documentation (Vazyme, Nanjing, China). The cDNA that was generated served as the foundational template for the subsequent RT-qPCR procedure. This RT-qPCR was executed utilizing the M5 HiPer Real-time PCR Super mix (Mei5 Biotech, Beijing, China). In order to normalize the levels of gene expression across the samples, the housekeeping gene *RhUBI2* was selected as an internal control. The quantification and analysis of gene expression data were meticulously carried out using the 2^−ΔΔCt^ methodological approach. To ensure the highest degree of accuracy in the results, each sample was subjected to testing in three distinct biological replicates, as well as three separate technical replicates. The specific primers that were employed for the RT-qPCR are comprehensively listed in Supplementary Table S3D.

### Yeast one-hybrid assay

The promoters of *RhGA3ox3, RhCESA8*, and RchiOBHmChr7g0187451, RchiOBHmChr3g0462711, RchiOBHmChr2g0106281, RchiOBHmChr6g0300381, RchiOBHmChr3g0468931, RchiOBHmChr2g016516 were inserted into pLacZ. *RhMYB70* was cloned into pB42AD, and the resulting plasmid was co-transformed with pLacZ into yeast EGY48. Yeast grown on SD/−Trp/−Ura medium, and then transformants were subsequently transferred to SD plates supplemented with galactose, raffinose, and X-β-gal. pB42AD-*RhMYB70* was co-transformed with empty pLacZ vectors to serve as negative controls. The combination of pB42AD-RhHB1 and pLacZ-*proRhGA20ox1* served as a positive control in the experiment [63]. Primer sequences are in Supplementary Table S3.

### Dual-luciferase reporter assay

The dual-luciferase assay adhered to the methodology outlined by Jing & Jin *et al* [30, 59]. A dual-luciferase reporter assay was utilized to assess the transcriptional activity of the RhMYB70. For the effector construct, the coding sequence of *RhMYB70* was amplified and inserted into the pBD vector. The empty pBD vector served as a negative control, whereas pBD-VP16 was employed as a positive control.

To generate reporter constructs for evaluating transactivation activity via a dual-luciferase reporter assay, the promoters of *RhGA3ox3* and *RhCESA8* were amplified and inserted upstream of the firefly luciferase (LUC) gene in the pGreenII 0800-LUC vector. Simultaneously, the coding sequence of *RhMYB70* was amplified and introduced into the pGreenII 62-SK vector.

The resultant effector and reporter constructs were introduced into the *Agrobacterium* strain GV3101, which contained the *pSoup* plasmid. *Agrobacterium* cultures carrying the effector and reporter constructs were mixed in equal volumes and co-infiltrated into the leaves of *N. benthamiana* plants. Three days postinfiltration, the leaves were harvested and luciferase activity was measured. The ratio of LUC activity to Renilla luciferase (REN) activity was computed using a Dual-Luciferase Reporter Assay System (Promega). Luminescence was quantified with a luminometer, and images illustrating the patterns of luciferase activity were captured using a CCD camera. The primer sequences used in the assay are provided in Supplementary Table S3.

### Chromatin immunoprecipitation-quantitative PCR

ChIP-qPCR was executed following the protocol detailed by Gong *et al*. [60]. Petals of roses that had been transiently overexpressing GFP-tagged proteins were cross-linked with a 1% (w/v) formaldehyde solution, which was then neutralized by the addition of glycine to a concentration of 0.125 M. Sonication was used to break the cross-linked chromatin, yielding DNA fragments between 400 and 750 bp in length. The GFP-tagged protein–DNA complexes were precipitated using GFP-Trap^®^ A at a dilution of 1:1000 (Chromotek, gtma-20). After an incubation period of 4°C overnight with the antibody, magnetic beads were applied to collect the complexes. The beads were washed extensively, and the DNA was extracted. The crosslinks were reversed by a 65°C incubation for 6 h, followed by DNA purification using a PCR purification kit. The purified DNA was subsequently assessed by qPCR with primers specific to various genomic regions. A list of the qPCR primers can be found in Supplementary Table S3.

### Cellulose content

Employing the Cellulose Content Assay Kit (BC4280, Solarbio Life Science Co., Ltd., Beijing, China), we quantified the cellulose in rose petals following the vendors’ specified procedures. We gathered petal samples from the natural states Stage 1, Stage 3, and Stage 5, as well as samples from various treatments at Stage 5: mock, GA_3_-treated, TRV and TRV-*RhMYB70,* TRV and TRV-*RhCESA8,* and TRV and TRV-*RhGA3ox3*. Prior to measurement, these samples underwent dehydration at 180°C to reach a consistent weight, followed by grinding into a fine powder. Precisely, 0.3 g of rose petal material was employed for the assay. Following the provided protocol, the spectrophotometer was set to a wavelength of 620 nm, using distilled water to calibrate the instrument to an absorbance baseline of 0 nm.

### Phylogenetic analysis

Amino acid sequence alignment was achieved using DNAMAN software. For phylogenetic studies, MEGA-X was employed to construct phylogenetic trees. The trees were bolstered by 1000 bootstrap replications to ensure the robustness of the phylogenetic inferences.

### Statistical analysis

All experimental procedures included a minimum of triplicate biological replications. The Student’s *t*-test was used for comparing two groups in the data analysis, while the Duncan’s test was utilized for comparisons among more than two groups. Significance was assigned to variations with a *P*-value < 0.05, indicating statistical relevance.

## Supplementary Material

Web_Material_uhaf134

## Data Availability

The transcriptome data were deposited in the NCBI Sequence Read Archive database under accession numbers PRJNA1162946. All data supporting the findings of this study are available within the paper and within its supplementary data published online.
