# Supplementary material for: The GAs–RhMYB70 feedback loop fine-tunes cell expansion and petal size by modulating cellulose content in rose
Source: Hortic Res. 2025 May 21;12(8):uhaf134. doi: 10.1093/hr/uhaf134 (PMC12268152; doi:10.1093/hr/uhaf134)
Supplement: Web_Material_uhaf134 [file web_material_uhaf134.zip › Supp_MS .pdf]

The GAs–RhMYB70 feedback loop fine-tunes cell expansion and petal size by modulating cellulose content in rose

Feifei Gong[2,+], Xiaoyu Wang[1,+], Qingcui Zhao[2,+], Dan Wang[1], Huijun Yan[1], Qigang Wang[1], Yiping Zhang[1], Yixin Zhang [1], Hongying Jian[1], Xianqin Qiu[1], Kaixue Tang[1], Hao Zhang[1], Weikun Jing[1,*]

1 Flower Research Institute of Yunnan Academy of Agricultural Sciences, Kunming, Yunnan, 650205, China

2 Beijing Key Laboratory of Development and Quality Control of Ornamental Crops, Department of Ornamental Horticulture, College of Horticulture, China Agricultural University, Beijing 100193, China

[+]These authors contributed equally to this work

*Author for correspondence: Weikun Jing (jwk093@163.com)

Supplementary figures and legends

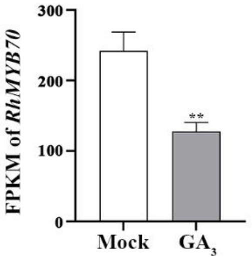

Supplementary Figure 1　The FPKM of *RhMYB70* in Mock and GA_3-treated petals.

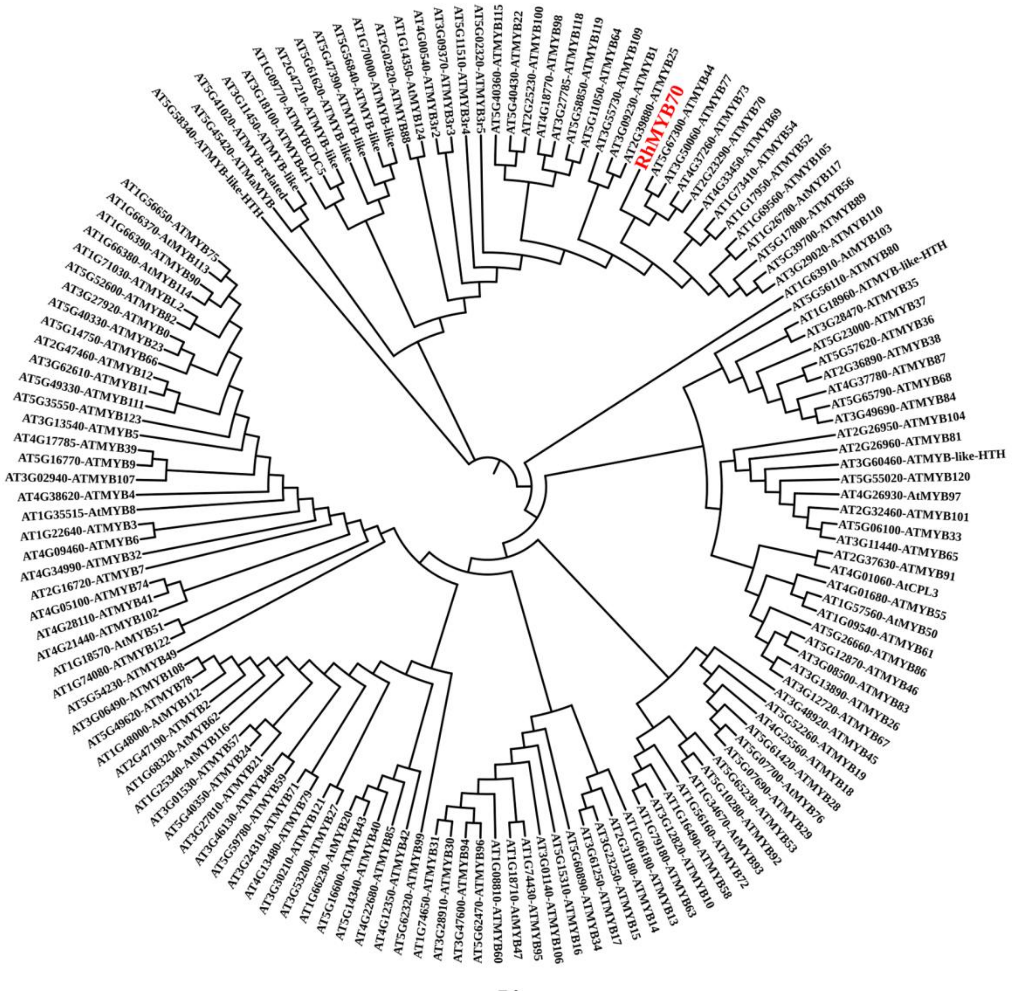

Supplementary Figure 2　Phylogenetic analysis of RhMYB70 and AtMYBs.

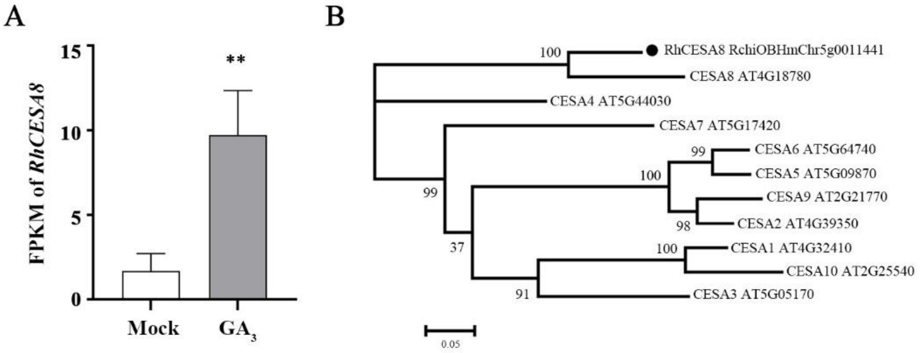

Supplementary Figure 3 Phylogenetic tree of RhCESA8 and AtCESAs.

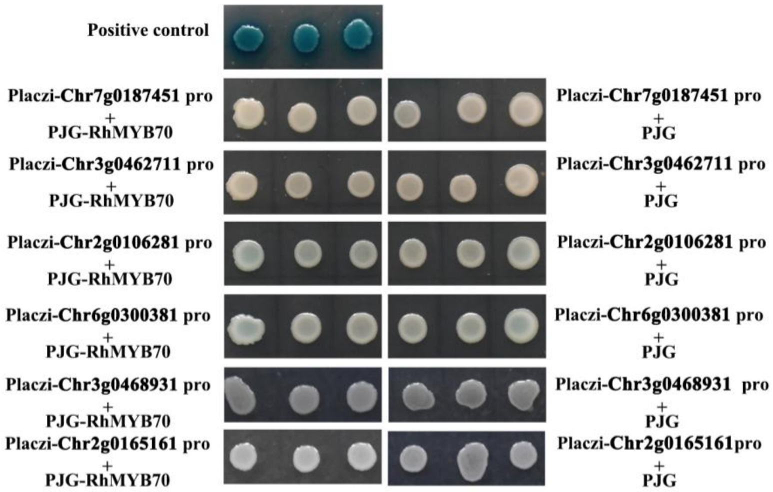

Supplementary Figure 4 Y1H revealing that RhMYB70 does not bind to the other DEGs promoters.

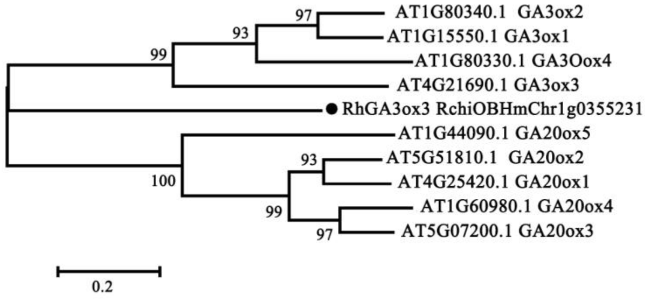

Supplementary Figure 5 Phylogenetic tree of RhGA3ox3.
